# Two *γ*‐Lactone Derivatives With Anti‐Inflammatory Activities From a Mangrove Endophytic Fungus *Alternaria* sp. HN‐17

**DOI:** 10.1002/cbdv.71654

**Published:** 2026-09-04

**Authors:** Yuru Sun, Bingbing Qian, Ruxue Mu, Zhongqian Xue, Linxuan Wu, Xin Huang, Yan Chen

**Affiliations:** ^1^ School of Pharmacy Anhui Medical University Hefei China

**Keywords:** *Alternaria* sp, anti‐inflammatory, endophytic fungus

## Abstract

Two *γ*‐lactone derivatives, alterlactones A‐B (**1–2**), along with one known analogue **3**, were isolated from the mangrove endophytic fungus *Alternaria* sp. HN‐17. Their structures were elucidated using comprehensive spectroscopic methods, including 1D and 2D NMR, HRESIMS, and electronic circular dichroism (ECD) calculations. Compounds **1** and **2** showed significant anti‐inflammatory activity with IC_50_ values of 10.2 and 13.5 µM, respectively.

## Graphical abstract

Two new γ‐lactone derivatives, alterlactones A–B (**1–2**), and a known analogue, **3**were isolated from *Alternaria* sp. HN‐17. Their structures were elucidated by NMR, HRESIMS and ECD calculations. Both **1** and **2** showed potent anti‐inflammatory activity.

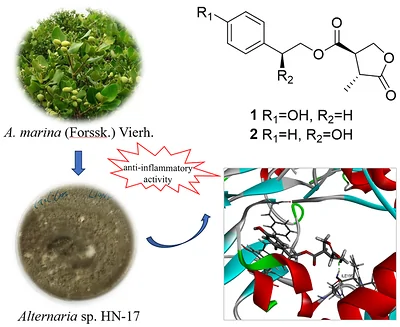

## 1 Introduction

Inflammation is a fundamental protective response that helps maintain tissue homeostasis during infection and injury. However, persistent or dysregulated inflammation is closely associated with the pathogenesis of many chronic diseases and has become a major therapeutic concern in modern medicine [1, 2]. Although currently available anti‐inflammatory and immunomodulatory therapies, including corticosteroids and biological therapeutics, have substantially improved disease management, their long‐term use is often limited by adverse effects, high costs, and variable or un‐sustained therapeutic efficacy [3]. Therefore, the discovery of safer and structurally novel anti‐inflammatory agents remains an important goal in drug research. In this context, natural products continue to serve as a valuable source of bioactive scaffolds for drug discovery, and have made major contributions to the development of new therapeutic agents [4, 5].

*Avicennia marina* (Forssk.) Vierh. is one of the most widely distributed mangrove species in tropical and subtropical coastal regions and has long attracted phytochemical and pharmacological interest as a medicinally valuable mangrove plant [6]. Mangrove ecosystems represent unique intertidal environments that harbor diverse microorganisms capable of producing structurally unusual and biologically active secondary metabolites. Among them, mangrove‐associated fungi, especially endophytic fungi, have attracted considerable attention as prolific sources of chemically diverse natural products, including polyketides with promising pharmacological potential [7, 8, 9, 10]. Nevertheless, the secondary metabolites of endophytic fungi associated with *A. marina* remain insufficiently explored. Previously, 15 new isocoumarin derivatives alterisocoumarins A–M were isolated from *Alternaria* sp. HN‐17, one strain from the fruit of *A. marina* [11]. Further exploration of the secondary metabolites for this strain, led to the isolation of two new *γ*‐lactone derivatives (**1–2**), together with one known analogue (**3**) [12]. Herein, we describe the isolation, structural elucidation, and spectroscopic characterization of these compounds based on comprehensive 1D and 2D NMR analyses and HRESIMS data.

## 2 Results and Discussion

**FIGURE 1:**
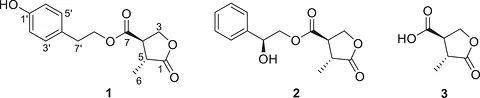
The structure of compounds **1–3**.

**FIGURE 2:**
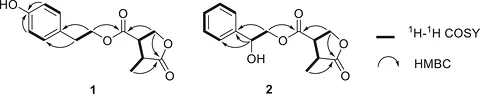
Key ^1^H–^1^H COSY and HMBC correlations for compounds **1** and **2**.

Compound **1** was obtained as white solid. The molecular formula C_14_H_16_O_5_Na was determined by HRESIMS at *m/z* 287.08900 [M + Na]^+^ (Figure S7). The ^1^H NMR spectrum (Table 1) showed one methyl signal at *δ*_H_ 1.30 (d, *J* = 7.1 Hz, 3H) and four aromatic proton signals at *δ*_H_ 6.78(d, *J* = 8.4 Hz, 2H) and 7.06(d, *J* = 8.4 Hz, 2H). The ^13^C NMR data (Table 1) revealed characteristic signals for one methyl group, three methylene groups, five methine groups (four olefinic), and two quaternary carbons (one carbonyl carbon). Comparison of the NMR spectra of **1** and **3** indicated that the structure of **1** contains a fragment of **3**. As shown in Figure 2, the ^1^H–^1^H COSY spin system of H_2_‐3/H‐4/H‐5/H_3_‐6, together with the HMBC cross‐peaks from H_2_‐3 to C‐1 and C‐7, and from H_3_‐6 to C‐1, established the presence of an *α*‐methyl paraconic acid subunit. In addition, the ^1^H–^1^H COSY correlations corresponding to H_2_‐7′/H_2_‐8′, H‐2′/H‐3′, and H‐5′/H‐6′, combined with HMBC cross‐peaks from H‐2′ and H‐6′ to C‐1′, from H_2_‐7′ to C‐3′, and from H_2_‐8′ to C‐5′, indicated a *p*‐hydroxyphenethyl alcohol moiety. This fragment was further confirmed to be attached at C‐7 via the HMBC correlation from H_2_‐8′ to C‐7. Thus, the planar structure of **1** was elucidated as shown in Figure 1.

**TABLE 1:** ^1^H and ^13^C (500/125 MHz) data for compounds **1** and **2**.

	1		2	
No	*δ*_C_, type	*δ*_H_ (*J* in Hz)	*δ*_C_, type	*δ*_H_ (*J* in Hz)
1	177.7, C		177.7, C	
3*α*	66.7, CH_2_	4.42, t (8.9)	66.7, CH_2_	4.50, t (8.0)
3*β*		4.18, t (9.4)		4.28, dd (7.7, 9.3)
4	48.1, CH	3.02, dd (9.8, 18.9)	47.9, CH	3.10, dd (8.5, 17.8)
5	37.9, CH	2.78, dq (7.1, 10.6)	37.9, CH	2.87, dt (7.2, 14.5)
6	14.6, CH_3_	1.30, d (7.1)	14.6, CH_3_	1.38, d (7.1)
7	170.5, C		174.6, C	
1'	154.6, C		128.1, CH	7.31, m
2', 6'	115.6, CH	6.78, brd (8.4)	128.6, CH	7.36, m
3', 5'	130.0, CH	7.06, brd (8.4)	126.1, CH	7.36, m
4'	129.1, C		140.3, C	
7'	34.1, CH_2_	2.89, t (6.8)	74.7, CH	4.84, d (8.0)
8'*α*	66.1, CH_2_	4.35, t (6.8)	68.0, CH_2_	3.78, d (11.5)
8'*β*				3.68, t (9.1)

Compound **2** was obtained as white solid, and the molecular formula was the same as **1**. The NMR data (Table 1) closely resembled those of **1**. The main difference was that the hydroxy group at C‐1' in **1** was replaced at C‐7' in **2**. The result was supported by the HMBC in arrows and COSY in solid lines (Figure 2). Thus, the planar structure of **2** was established.

**FIGURE 3:**
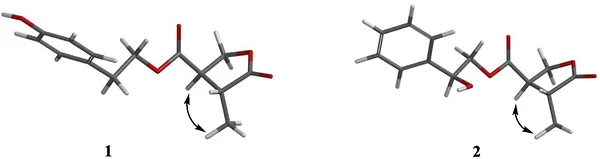
The key NOESY correlations for compounds **1–2**.

**FIGURE 4:**
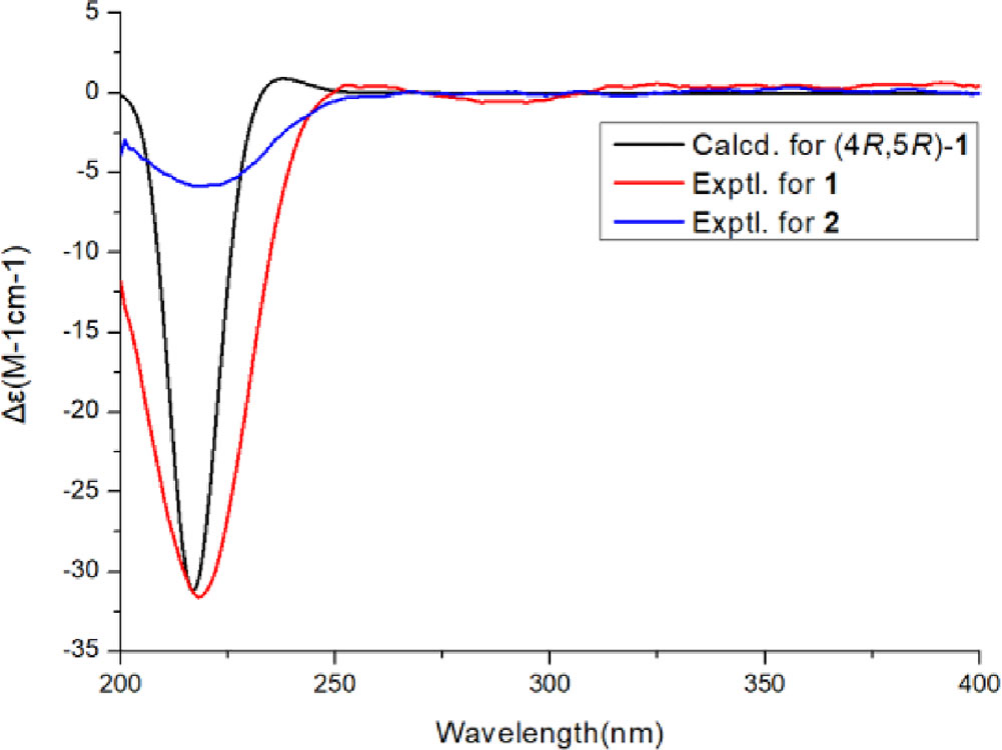
Comparison of the measured and calculated ECD spectra for compounds **1** and **2**.

The relative configuration of **1** and **2** was assigned by analysis of the NOESY spectrum (Figure 3). The correlations between H‐4/H_3_‐6 indicated that these protons are co‐facial. Then, the absolute configuration of **1** and **2** was determined as 4*R*, 5*R* based on the good agreement of its experimental ECD spectrum (Figure 4) with that calculated for (4*R*,5*R*)‐**1**. To establish the absolute configuration at C‐7' in compound **2**, it was hydrolyzed to afford compound **3** and 1‐phenyl‐1,2‐ethanediol (Figures S15–S17). The specific rotation of 1‐phenyl‐1,2‐ethanediol was determined as ${\left[\alpha \right]}_{D}^{25}$+10.4 (*c* 0.26, MeOH). By comparison with the reported values for known chiral compounds, including phomoaspardiol (${\left[\alpha \right]}_{D}^{21}$−28.5, *c* 0.07, MeOH) [13], (1*S*)‐(4‐acetylphenyl)‐1,2‐ethanediol (${\left[\alpha \right]}_{D}^{16}$+8.7, *c* 0.50, MeOH) [14], and (*S*)‐(+)‐2‐(3,4‐dihydroxyphenyl)‐2‐ethoxyethanol (${\left[\alpha \right]}_{D}^{25}$+32.0, *c* 0.005, CHCl_3_) [15], the 7'*S* configuration in compound **2** was unambiguously assigned.

**FIGURE 5:**
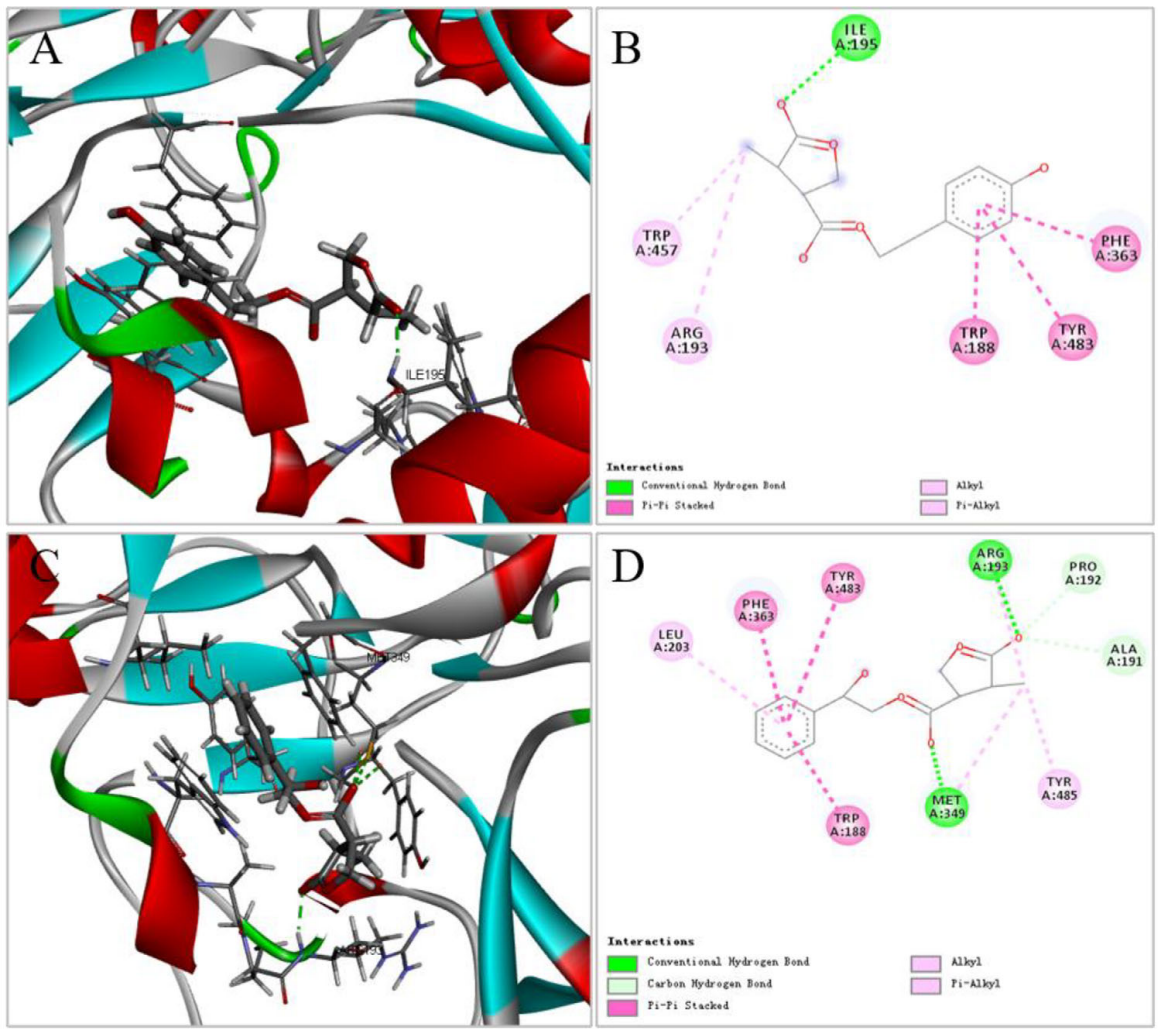
Compound **1** (A) and **2** (C) docked to iNOS (PDB code: 3HR4). 2D interaction diagrams of compound **1** (B) and **2** (D).

The inhibitory activity of compounds **1–3** against NO release in LPS‐induced RAW 264.7 cells were measured. Compounds **1–2** showed significant anti‐inflammatory activity with IC_50_ values of 10.2 and 13.5 µM, respectively. Compound **3** showed moderate activity with IC_50_ value of 20.6 µM. None of the compounds showed obvious cytotoxicity at the effective concentrations (Figures S18 and S19). The positive control was *N* ^ω^‐methyl‐_L_‐arginine (L‐NMMA) (IC_50_: 17.7 µM). To elucidate the underlying mechanism of bioactive compound and protein, a molecular docking study was performed between the target compounds **1–2** and inducible NOS (iNOS). As shown in Figure 5A,B, one hydrogen bond was formed between carbonyl group of **1** and the backbone amine ILE195 in iNOS, with the binding energy of − 7.29 kcal/mol. Two hydrogen bonds were formed between the carbonyl group of **2** and Arg193, Met349 in iNOS, with the binding energy of − 9.96 kcal/mol (Figure 5C,D). In addition to hydrogen bonding forces, the docking results also demonstrated the carbon‐hydrogen bonds, π–π stacking interactions, and alkyl/*π*‐alkyl interactions between the compound and the protein. The results showed that compounds **1–2** binds deeply in the active cavity of iNOS.

## 3 Conclusions

In summary, two new *γ*‐lactone derivatives, compounds **1** and **2**, along with one known analogue **3**, were isolated from the mangrove endophytic fungus *Alternaria* sp. HN‐17. Compounds **1** and **2** showed significant anti‐inflammatory activity with IC_50_ values of 10.2 and 13.5 µM, respectively. The molecular docking results show that this compound exhibits a strong binding effect on iNOS. The results indicate that these *γ*‐lactones are potential anti‐inflammatory lead compounds. *γ*‐Lactone is a core scaffold of many natural products, and derivatives with this structural fragment exhibit a wide range of biological activities, including anti‐inflammatory [12], cytotoxic [17], neuroprotective [18], antimicrobial [19, 20], dehalogenation [21], and anti‐*Trypanosoma cruzi* [22] activity. Further structural optimization and in‐depth mechanistic as well as in vivo studies of *γ*‐lactone derivatives will be carried out to explore their full potential as novel drug candidates.

## 4 Experimental Section

### 4.1 General Experimental Procedures

General experimental procedures were the same as those reported previously [23].

### 4.2 Fungal Material and Culture

The source of the strain and the fermentation extraction method are the same as those described previously [11]. Solid rice medium (containing 80 g of raw rice and 70 mL of 0.3% seawater) is used for the fermentation cultivation of the strain. The fermentation broth underwent triple extraction with ethyl acetate, yielding 55.8 g of crude extract. The components were eluted using a gradient of petroleum ether/ethyl acetate (10% to 100% v/v) to yield 10 fractions (Fr.1 to Fr.10). Fr.4 was separated by silica gel chromatographic column (CC, 200 mesh silica) eluting by silica gel CC (CH_2_Cl_2_/MeOH v/v, 83:17), and further purified by Sephadex LH‐20 with CH_2_Cl_2_/MeOH (v/v, 1:1) to yield compound **1** (5.2 mg). Compounds **2** (8.6 mg) and **3** (3.5 mg) were obtained by silica gel CC eluting with CH_2_Cl_2_/MeOH (87:13, v/v) from Fr.6.

Alterlactone A (**1**): white solid; ${\left[\alpha \right]}_{D}^{25}$ = −37.2 (*с* 0.32, MeOH); UV (MeOH) λ_max_ (log ε): 210 (3.35), 258 (3.13), 300 (3.0) nm; IR (KBr) *ν*_max_: 3035, 2970, 2876, 1756, 1736, 1645, 1310, 1020 cm^−1^; ^1^H and ^13^C NMR data (500 MHz, CDCl_3_): Table 1; HRESIMS m/z 287.08900 [M + Na]^+^ (calcd. for C_14_H_16_O_5_Na, 287.08902)

Alterlactone B (**2**): white solid; ${\left[\alpha \right]}_{D}^{25}$ = −26.5 (*c* 0.25); UV (MeOH) λ_max_ (log ε): 205 (3.34), 258 (3.10), 300 (3.02) nm; IR (KBr) *ν*_max_: 3038, 2968, 2880, 1761, 1735, 1630, 1316, 1018 cm^−1^; ^1^H and ^13^C NMR (500 MHz, CDCl_3_): Table 1; HRESIMS m/z 287.08894 [M + Na]^+^ (calcd. for C_14_H_16_O_5_Na, 287.08896).

### 4.3 Quantum Chemical Calculations

The quantum chemical calculations were carried out by the method outlined previously [24, 25]. Brief, Molecular Merck force field (MMFF) and density functional theory/time‐dependent density functional theory (DFT/TD‐DFT) calculations were performed using Spartan’14 software (Wavefunction Inc.) and Gaussian 09 program, respectively. Conformers with a Boltzmann distribution above 10% were selected for ECD calculations. Conformer generation and geometry optimization were carried out via DFT calculations at the B3LYP/6‐31G(d) level in methanol with the CPCM conductor‐like polarizable continuum model. Theoretical calculations of the ECD spectrum for compound 1 were performed in methanol using TD‐DFT at the B3LYP/6‐31G level. The theoretical ECD spectrum was generated using SpecDis 1.6 (University of Würzburg) and OriginPro 8.5 (OriginLab Ltd.) with Gaussian band shapes at *σ* = 0.30 eV.

### 4.4 Cell Culture, Cell Viability, and NO Inhibition Assay

Mycoplasma‐free RAW 264.7 mouse monocyte/macrophage leukemia cells (Servicebio, Wuhan, China) were cultured in RAW 264.7‐specific medium (Pricella Biotechnology, Wuhan, China) at 37°C in a humidified atmosphere containing 5% CO_2_. Cell viability was determined using the CCK‐8 assay as previously described [26]. Briefly, the cells were treated with varying concentrations (3.125–50 µM) of test compounds or L‐NMMA for 24 h. For nitric oxide (NO) determination, RAW264.7 cells were seeded into 24‐well plates under identical conditions and exposed to a range of compound concentrations in combination with LPS (1 µg/mL) for 24 h. Finally, the absorbance was measured at 540 nm using a microplate reader. The NO inhibition rate was calculated using the formula: NO inhibition rate = [(OD_(_LPS_)_ − OD_(_control_)_) − (OD_(_compound_)_ − OD_(_control_)_)] / (OD_(_LPS_)_ − OD_(_control_)_) × 100% to evaluate the anti‐inflammatory activity. Data are mean ± SD, *n* = 3 biological replicates. Statistical analysis was performed by one way ANOVA with post hoc test: ns, not significant; *p < 0.05; ***p < 0.001; ***p < 0.0001 versus LPS control group.

### 4.5 Molecular Docking Studies

The molecular docking study was accomplished by Sybyl‐X 2.0 [27], and crystal structure of iNOS (PDB: 3HR4) was obtained from the RCSB Protein Data Bank. Docking of compounds **1** and **2** were first optimized using the Gaussian 09 program with DFT calculations at the B3LYP/6‐31G(d) level. All polar hydrogen atoms were added and solvation parameters were assigned. Before the docking process, the ligand substructures were extracted and water molecules were removed. The surflex‐dock total score was expressed in – Log (Kd) to represent binding affinities.

### 4.6 Hydrolysis and Methyl Esterification Reaction

A solution of **2** (6.8 mg) in THF (tetrahydrofuran, 1.5 mL) was stirred with NaOH solution (1.0 M, 1.0 mL) at room temperature for 10 h. Then, the mixture was extracted with EtOAc, and the organic solution was evaporated under reduced pressure to yield the 1‐phenyl‐1,2‐ethanediol (3.8 mg, Figures S15 and S16). While, the aqueous layer was acidified by HCl solution (1.0 M), and extracted with EtOAc. The organic solution was concentrated to give compound **3** (4.0 mg, Figures S13 and S14).

## Author Contributions

**Yuru Sun**: writing – fermental cultivation, article writing, and data curation. **Bingbing Qin**: writing – fermental cultivation and data curation. **Rbuxue Mu**: activity screening. **Zhongqian Xue**: separation and data curation. **Linxuan Wu and Xin Huang**: separation. **Yan Chen**: writing – review & editing, supervision, and project administration.

## Conflicts of Interest

The authors declare no conflicts of interest.

## Supporting information




**Supporting File 1**: cbdv71654‐sup‐0001‐SuppMat.docx

## Data Availability

The data that support the findings of this study are available in the supplementary material of this article.
