# Supplementary material for: Two γ‐Lactone Derivatives With Anti‐Inflammatory Activities From a Mangrove Endophytic Fungus Alternaria sp. HN‐17
Source: Chem Biodivers. 2026 Sep 4;23(9):e71654. doi: 10.1002/cbdv.71654 (PMC13543768; doi:10.1002/cbdv.71654)
Supplement: Supplementary file 1 [file CBDV-23-e71654-s001.docx]

**Supplementary Material**

**Two *γ*-lactone derivatives with Anti-inflammatory activities from a Mangrove Endophytic Fungus *Alternaria* sp. HN-17.**

Yuru Sun^⊥^, Bingbing Qian^⊥^, Ruxue Mu, Zhongqian Xue, Linxuan Wu, Xin Huang, Yan Chen*

School of pharmacy, Anhui Medical University, Hefei 230032, China

Correspondence author: cychemistry@ahmu.edu.cn (Y. Chen)

Author Contributions

^⊥^Yuru Sun and Bingbing Qian contributed equally.

**Supporting Information Contents:**

**Figure S1.** ^1^H NMR spectrum of compound **1** (500 MHz, CDCl_3_).

**Figure S2.** ^13^C NMR spectrum of compound **1** (125 MHz, CDCl_3_).

**Figure S3.** HSQC spectrum of compound **1**.

**Figure S4.** ^1^H-^1^H COSY spectrum of compound **1**.

**Figure S5.** HMBC spectrum of compound **1**.

**Figure S6.** HRESIMS spectrum of compound **1**.

**Figure S7.** ^1^H NMR spectrum of compound **2** (500 MHz, CDCl_3_).

**Figure S8.** ^13^C NMR spectrum of compound **2** (125 MHz, CDCl_3_).

**Figure S9.** HSQC spectrum of compound **2**.

**Figure S10.** ^1^H-^1^H COSY spectrum of compound **2**.

**Figure S11.** HMBC spectrum of compound **2**.

**Figure S12.** HRESIMS spectrum of compound **2**.

**Figure S13.** ^1^H NMR spectrum of compound **3** (500 MHz, CDCl_3_).

**Figure S14.** ^13^C NMR spectrum of compound **3** (125 MHz, CDCl_3_).

**Figure S15.** ^1^H NMR spectrum of 1-Phenyl-1,2-ethanediol (500 MHz, CDCl_3_).

**Figure S16.** ^13^C NMR spectrum of 1-Phenyl-1,2-ethanediol (125 MHz, CDCl_3_).

**Figure S17.** The hydrolysis reaction of compound **2**.

**Figure S18.** Cell viability of RAW 264.7 cells treated with compounds **1–3** at concentrations of 3.125–50 μM for 24 h, determined by CCK-8 assay. Data are mean ± SD, n = 3 biological replicates.

**Figure S19.** Dose‑response curves of NO inhibition in RAW 264.7 cells treated with compounds **1–3** (3.125–50 μM, 24 h), measured by Griess reagent. Data are mean ± SD, n = 3 biological replicates. Statistical analysis was performed by one‑way ANOVA with post‑hoc test: ns, not significant; **p* < 0.05; ****p* < 0.001; ****p < 0.0001* vs. LPS control group.

Figure S1. ^1^H NMR spectrum of compound **1** (500 MHz, CDCl_3_)

Figure S2. ^13^C NMR spectrum of compound **1** (500 MHz, CDCl_3_)

Figure S3. HSQC spectrum of compound **1**.

Figure S4.^1^H-^1^H COSY spectrum of compound **1**

Figure S5. HMBC spectrum of compound **1**.

Figure S6. NOESY spectrum of compound **1**.

Figure S7. HRESIMS spectrum of compound **1**

Figure S8. ^1^H NMR spectrum of compound **2** (500 MHz, CDCl_3_)

Figure S9. ^13^C NMR spectrum of compound **2** (500 MHz, CDCl_3_)

Figure S10. HSQC spectrum of compound **2**

Figure S11. ^1^H-^1^H COSY spectrum of compound **2**

Figure S12. HMBC spectrum of compound **2**.

Figure S13. NOESY spectrum of compound **2**.

Figure S14. HRESIMS spectrum of compound **2**.

**Figure S13.** ^1^H NMR spectrum of compound **3** (500 MHz, CDCl_3_).

**Figure S14.** ^13^C NMR spectrum of compound **3** (125 MHz, CDCl_3_).

**Figure S15.** ^1^H NMR spectrum of 1-Phenyl-1,2-ethanediol (500 MHz, CDCl_3_).

**Figure S16.** ^13^C NMR spectrum of 1-Phenyl-1,2-ethanediol (125 MHz, CDCl_3_).

**Figure S17.** The hydrolysis reaction of compound **2**.

**Figure S18.** Cell viability of RAW 264.7 cells treated with compounds **1–3** at concentrations of 3.125–50 μM for 24 h, determined by CCK-8 assay. Data are mean ± SD, n = 3 biological replicates.

**Figure S19.** Dose‑response curves of NO inhibition in RAW 264.7 cells treated with compounds **1–3** (3.125–50 μM, 24 h), measured by Griess reagent. Data are mean ± SD, n = 3 biological replicates. Statistical analysis was performed by one‑way ANOVA with post‑hoc test: ns, not significant; **p* < 0.05; ****p* < 0.001; ****p < 0.0001* vs. LPS control group.
